# Direct Immersion–Solid Phase Microextraction for Therapeutic Drug Monitoring of Patients with Mood Disorders

**DOI:** 10.3390/molecules29030676

**Published:** 2024-01-31

**Authors:** Magdalena Świądro-Piętoń, Dominika Dudek, Renata Wietecha-Posłuszny

**Affiliations:** 1 Laboratory for Forensic Chemistry, Department of Analytical Chemistry, Faculty of Chemistry, Jagiellonian University, 2, Gronostajowa St., 30-387 Kraków, Poland; mg.swiadro@op.pl; 2 Department of Adult Psychiatry, Medical College, Jagiellonian University, 21a, Mikołaja Kopernika St., 31-387 Kraków, Poland; dominika.dudek@poczta.fm

**Keywords:** DI-SPME method, mood disorder, therapeutic drug monitoring

## Abstract

This article discusses a new method for monitoring drug concentrations in blood samples from patients with mood disorders. The method uses solid-phase microextraction to extract analytes directly from blood samples. It has been adapted to identify the most commonly used drugs in mood disorders, including amitriptyline, citalopram, fluoxetine, paroxetine, sertraline, trazodone, duloxetine, venlafaxine, lamotrigine, quetiapine, olanzapine, and mirtazapine. The analysis is carried out using high-performance liquid chromatography coupled with mass spectroscopy. The proposed DI-SPME/LC-MS method allows for a simple and quick screening analysis while minimizing the volume of the tested sample and solvent, in line with the principles of green analytical chemistry. The method was used to analyze 38 blood samples taken from patients with mood disorders, and drug concentrations were determined and compared with therapeutic and toxic dose ranges. This allowed for better control of the course of treatment.

## 1. Introduction

Mood disorders, including depression and bipolar disorders, are increasingly becoming a public health problem in terms of their prevalence and morbidity. Symptoms of affective disorders not only persist for a long time but are also highly likely to recur. The most important treatment for depressive symptoms is pharmacotherapy with antidepressants. The treatment of mood disorders involves several phases, and it is intensive and long-lasting to prevent relapses. The medications most often used in the case of mood disorders are selective serotonin reuptake inhibitors, tricyclic antidepressants, double serotonergic antidepressants, serotonin, and norepinephrine reuptake inhibitors, and others like anticonvulsants and atypical neuroleptics [1]. Hence, the treatment of people affected by depressive and bipolar disorders is increasingly being monitored [2,3]. Therapeutic drug monitoring (TDM) involves measuring the concentration of certain drugs in biological materials like blood, plasma, and serum. TDM is used to personalize, optimize, and ensure the safety and effectiveness of treatment by assessing drug levels [4,5].

The TDM process involves evaluating the concentration of drugs present in the biological material being analyzed. To do this, we isolate the analytes to be tested from the matrix. This is carried out using various extraction techniques such as microwave-assisted extraction [6], solid-phase extraction [7], or the dried blood spot method coupled with ultrasound-assisted extraction [8,9]. An interesting alternative is the use of solid-phase microextraction (SPME), which is a quick and simple sample preparation technique. In this technique, fibers coated with a suitable material are used to isolate and concentrate analytes from the sample. Microextraction is carried out by allowing an equilibrium of analytes between the sample matrix and the extraction coating. After the adsorption phase, the fiber coating is desorbed by placing SPME fibers in a desorption solvent to free the analytes [10,11].

There are three types of SPME extraction methods. The first one is direct immersion–solid-phase microextraction (DI-SPME), where the fibers are directly introduced into the sample. In this method, the analytes pass from the sample matrix to the fiber extraction coating [12,13]. The second method is headspace–solid-phase microextraction (HS-SPME), which is used when examining samples containing volatile analytes. In this method, the fiber is placed over the aqueous sample matrix [14]. The third method is membrane–solid-phase microextraction (M-SPME), which involves membrane protection. This method is used for samples with high-molecular-weight interfering substances, or nonvolatile analytes for better precision and reproducibility [15].

The SPME technique is a highly advantageous method for drug analysis. One of its key benefits is its simplicity, which allows for the easy isolation and identification of multiple drugs in a complex biological matrix with minimal sample preparation. This is achieved by using a small fiber coated with a stationary phase, which selectively extracts the target analytes from the sample matrix [16].

Another advantage of the SPME method is its compliance with the principles of green chemistry. This is due to the minimal volumes of samples and solvents required, which reduces the environmental impact of the procedure. Additionally, the cost of the procedure is also reduced as a result of the minimal use of solvents and the possibility of multiple uses of the fibers [17]. Overall, the SPME technique is a highly efficient and cost-effective method for drug analysis, which also promotes environmentally friendly practices.

Ensuring patients comply with recommended treatment plans and take their prescribed medication is crucial for achieving the desired therapeutic effect. To this end, a research study was conducted to develop a methodology that can effectively monitor the concentration of drugs from different groups, including selective serotonin reuptake inhibitors and tricyclic antidepressants. The study utilized the DI-SPME/LC-MS methodology to analyze blood samples taken from a group of patients with mood disorders. This innovative method is the first of its kind that allows for the isolation of drugs used in the treatment of mood disorders while adhering to the principles of green chemistry. The methodology minimizes procedures and reduces the volume of solvents used, making it an environmentally friendly option. The results of this study have significant implications for the development of more effective and sustainable drug-monitoring methods in the future.

## 2. Results and Discussion

### 2.1. I-SPME Extraction

The research initially involved testing the DI-SPME extraction process suggested by Majda et al. [12]. This process is explained in detail in Section 3.3. To begin, drug standards and deuterated internal standards were prepared by diluting the starting solutions to gain a concentration of 100 ng/mL. The researchers then tested if the DI-SPME method, in combination with the proposed LC-MS operating parameters, could extract and determine specific drugs including AMI, CIT, FLU, PAR, SER, TRA, DUL, MIR, VEN, LAM, QUE, and OLA.

**Figure 1:**
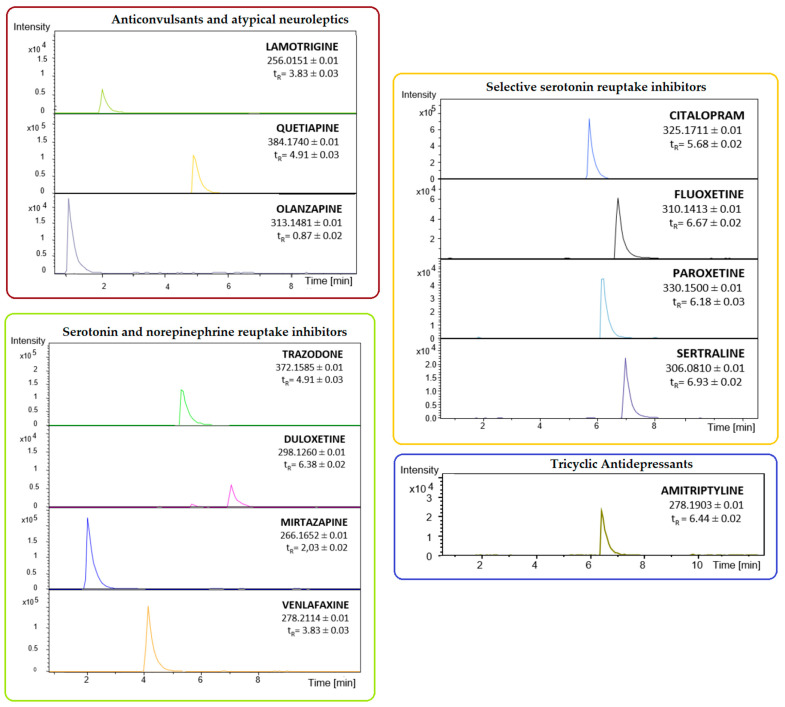
The chromatograms confirming the detection of the only drug used in the pharmacotherapy of mood disorders—amitriptyline, aripiprazole, carbamazepine, citalopram, clomipramine, desipramine, flunitrazepam, fluoxetine, fluvoxamine, imipramine, ketamine, paroxetine, sertraline, trazodone, duloxetine, mirtazapine, nortriptyline, lamotrigine, quetiapine, and olanzapine at a concentration of 250 ng/mL—after application of the proposed DI-SPME/LC-MS methodology.

As shown in Figure 1, the DI-SPME/LC-MS methodology successfully made it possible to isolate all the labeled drugs. The chromatograms were characterized by a good separation of all analytes, and with no double peaks. Each signal indicates a decent level of intensity. Additionally, the analytes detected exhibited different retention times, which will offer a more comprehensive understanding of the patient’s sample composition—see Table 1. Additionally, the structure of analyzed drugs is provided in Supplementary Materials as Figure S1.

Table 1 compiled a comprehensive list of all analytes used in research, along with their corresponding assigned internal standards. Additionally, the monitored ion mass range [M + H]^+^ and detected retention time for each analyte are also provided in the table for further reference and analysis.

### 2.2. Validation Process

During the research, a validation procedure was conducted to validate the accuracy and reliability of the results. The linearity was determined to ensure that the response from the instrument was proportional to the concentration of the drug being analyzed. The lower limit of quantification (LLOQ) and detection (LOD) were also determined to ensure that the instrument could detect and quantify the drug at low concentrations. Additionally, the precision of the results obtained during the day and between days was analyzed to determine the consistency and reliability of the test. In addition to the initial analysis, a series of tests were conducted to ensure the accuracy and reliability of the results. These tests included carryover, dilution integrity, and selectivity tests, all of which were designed to demonstrate that the drug being analyzed could be detected even in the presence of other substances. The results of all these validation parameters have been compiled and are presented in Table 2.

The results of the validation parameters obtained from the analysis indicate that there is a strong linear relationship between the analytical signal obtained and the concentration of the analytes in the blood sample. In fact, for most of the substances tested, the coefficient of determination R^2^ of the determined curves was greater than 0.98, which indicates a high degree of accuracy in the testing methodology. The only substances that had a lower value of the coefficient R^2^ were MIR, LAM, and OLA, which were still at a satisfactory level of above 0.96. Additionally, the calculated LOD (limit of detection) values and LLOQ (limit of quantification) values range from 0.14 to 4.29 [ng/mL] and 0.70–21.47 [ng/mL], respectively. These values are at least one order of magnitude lower than the theoretical therapeutic dose values, which suggests that the proposed methodology is highly effective in determining drug concentrations at both therapeutic and toxic levels.

The proposed methodology was found to be effective and precise in determining drug concentrations. Specifically, the precision values for the lowest concentration sample (50 ng/mL) did not exceed 20%, while samples with concentrations of 150 and 250 ng/mL had precision values of 15%. These results meet the required criteria, indicating the accuracy of the proposed methodology. Despite lower determination coefficients R^2^, the tested drugs (AMI, CIT, FLU, PAR, and VEN) showed lower LOD and LOQ values than those published in the article by Majda et al. [12]. However, the coefficients of variation determined in the proposed study were higher than in Majda’s work. Another study conducted by Świądro et al. [8] aimed to select the best extraction technique for the isolation of antidepressants, and found lower R^2^ coefficients and higher LOD and LLOQ values for compounds CIT, FLU, PAR, SER, and VEN, except for the LLOQ for SER. Precision values during the day and between days for CIT, PAR, and VEN were also higher in the proposed studies.

Table 2 presents the final results for the matrix effects, which indicates the influence of matrix components on the ionization process of analytes in the MS ion source. It is noteworthy that the ME results are lower than 15%, implying that the matrix components have no or minimal impact on the ionization process of the analytes.

**Figure 2:**
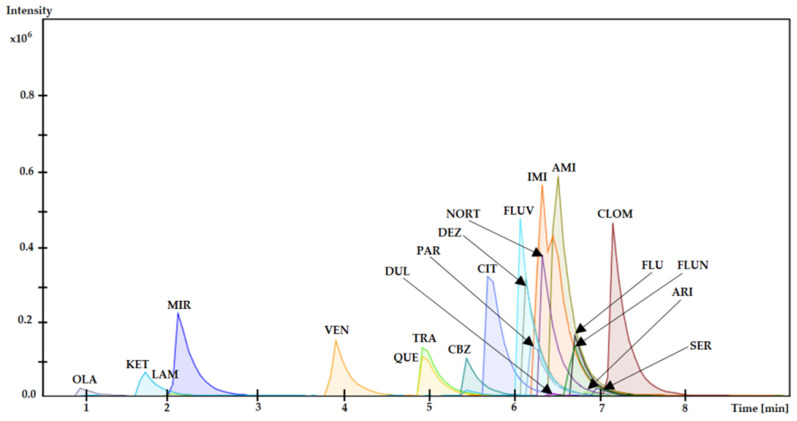
The chromatogram obtained as a result of the analysis confirms the selectivity of the optimized DI-SPME/LC-MS method.

The study also examined carryover, dilution integrity, and selectivity. An analysis of the results revealed no changes due to carryover. The optimized method is resistant to sample dilution, and dilution integrity was achieved with satisfactory precision. Moreover, the optimized method is also highly selective, as we observed no influence between the analyzed drugs and ketamine (KET) and flunitrazepam (FLUN), which are the main ingredients of the “date rape drug”, and other drugs—carbamazepine (CBZ), desipramine (DEZ), nortriptyline (NORT), imipramine (IMI), fluvoxamine (FLUV), aripiprazole (ARI) and clomipramine (CLOM)—less frequently used in the pharmacotherapy of metabolic disorders. The obtained result was shown as a chromatogram, from which we may see that there is no interference between all analyzed substances—Figure 2.

Moving forward, the optimized DI-SPME/LC-MS methodology may be applied to further analyze patient samples. Our validation process has confirmed the effectiveness of this approach, and we are confident that it will yield valuable insights in our ongoing research.

### 2.3. Analysis of Patient Samples

**Figure 3:**
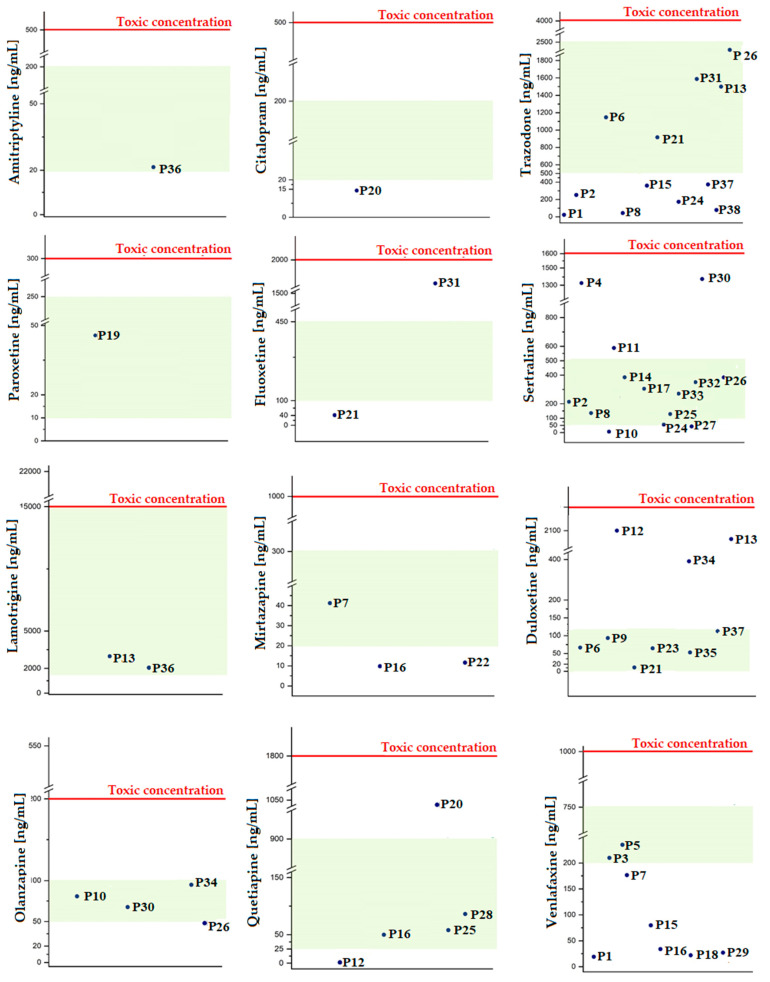
Summary of results obtained from DI-SPME/LC-MS analysis of real samples, including the therapeutic range (marked as a green area) and toxic (red line).

The results obtained in the validation process confirm the effectiveness of the proposed methodology in the qualitative and quantitative analysis of a group of drugs used in the treatment of mood disorders. Therefore, in the last stage, the developed DI-SPME/LC-MS method was used to analyze blood samples collected intravitally from patients. Before chromatographic measurement, 90 µL of a mixture of standard substances was added to the samples. They were then subjected to DI-SPME extraction and LC-MS analysis, analogously as described in Section 3.3. The results obtained for the determination of AMI, CIT, FLU, PAR, SER, TRA, DUL, MIR, VEN, LAM, QUE, and OLA in patient samples (P_1–P_38) are summarized in Figure 3, marking the therapeutic and toxic range for the analytes determined.

The most frequent preparation taken by patients contained TRA, SER, VEN, and DUL—see Figure 3 and Table 2. Fourteen patients took SER, most often once a day, in the morning or evening, mainly at a dose of 50 or 100 mg. The determined SER concentrations in patient samples are within the therapeutic range. In the case of P_4, P_11, and P_30, the drug level indicates that the patients probably took medications before blood collection. However, the obtained concentrations of the tested drugs do not exceed the toxic range.

In the case of TRA, twelve patients took the drug in the morning at a dose of 50, 75, or 150 mg. In the group of patients (P_6, P_21, and P_31) who took TRA in the evening at a dose of 150 mg, the determined concentrations of this drug were within the therapeutic range. Six patients were taking VEN in the morning at a dose of 75 or 150 mg. In all cases, the concentration of drugs taken was below the therapeutic window.

The MIR content in one of three patient samples was within the therapeutic range, and, in two samples, it was below the therapeutic dose. A similar situation, in which the obtained concentrations are below the therapeutic window, can be noted in patient P_20, who used drugs containing CIT. Moreover, a similar case was observed in the group of patients using DUL. In six samples, the DUL concentrations obtained were within the therapeutic range. Only for samples P_12, P_13, and P_34 did the obtained level exceed the limits of the therapeutic range several times and even approached the toxic range.

Normal levels that fall within the therapeutic range are also marked in the group of patients using AMI (P_36), OLA (P_10, P_26, P_30 and P_34), LAM (P_13 and P_36), and PAR (P_19). In the case of FLU determination, the concentration in the sample (P_31) exceeded the therapeutic range, while, in the sample (P_21), it was below the therapeutic window.

Analyzing the obtained results, it can be seen that most of the determined concentration levels are below the lower limit of the therapeutic dose range. However, attention should be paid to the biological half-life of the analytes, the time of taking the last dose of the drug, and the time of collecting samples for testing. If blood is obtained in the morning just before taking the next tablet, the level of the analytes in the blood may be low, depending on the metabolism and excretion of the drug from the body. The concentration that will produce a clinical effect in a given patient depends on individual characteristics such as age, metabolism, comorbidities, as well as interactions with other drugs or substances such as cigarettes or alcohol. There is also a risk of some non-compliance between patient practice and therapeutic recommendations. This means that, despite the indications to take one tablet a day, the patient takes several or skips a dose. All these factors may influence fluctuations in the analyte concentration in the patient’s body, which directly translates into the clinical effect. On the other hand, it should be remembered that concentrations within the therapeutic window should not be sought if the dose the patient receives produces the desired clinical effect while minimizing side effects as best as possible. Nevertheless, the results from real samples confirm the potential of the newly developed DI-SPME/LC-MS method in the therapeutic drug monitoring of patients with diagnosed mood disorders.

## 3. Materials and Methods

### 3.1. Chemicals and Laboratory Equipment

Standard solutions of analytes and internal standards at concentration of 1 mg/mL—amitriptyline (AMI), aripiprazole (ARI), carbamazepine (CBZ), citalopram (CIT), clomipramine (CLOM), desipramine (DEZ), flunitrazepam (FLUN), fluoxetine (FLU), fluvoxamine (FLUV), imipramine (IMI), ketamine (KET), paroxetine (PAR), sertraline (SER), trazodone (TRA), duloxetine (DUL), mirtazapine (MIR), nortriptyline (NORT), venlafaxine (VEN), lamotrigine (LAM), quetiapine (QUE), olanzapine (OLA), amitriptyline_d_3_ (AMI_d_3_), fluoxetine_d_6_ (FLU_d_6_), paroxetine_d_6_ (PAR_d_6_), venlafaxine_d_6_ (VEN_d_6_), carbamazepine_d_10_ (CBZ_d_10_)—in methanol were also purchased from Lipomed AG (Arlesheim, Switzerland). All standard drug stock solutions (10 μg/mL) used in the experiment were prepared in methanol and stored at −20 °C.

The SPME experiment was conducted using commercially available C-18 SPME-LC silica fibers (Supelco, Bellefonte, PN, USA), coating thickness: 45 µm, and fiber coverage length: 1.3 mm, supplied by Merck (Darmstadt, Germany). The following VWR devices (Randor, PA, USA) were also utilized: HPLC vials (1.5 mL), inserts (200 µL), Thermal Shake Touch, and Digital Vortex Mixer. Concentrator Plus was purchased from Eppendorf AG (Hamburg, Germany). Adjustable manual pipettes were supplied by Sartorius AG (Göttingen, Germany).

The reagents such as sodium hydroxide, methanol, acetonitrile, and isopropyl alcohol, used in the experiment, were purchased from Fluka Analytical (Seelze, Germany). The analytical-grade sodium hydroxide, isopropyl alcohol, and ammonium formate were supplied by Sigma–Aldrich (St. Louis, MA, USA), whereas formic acid was purchased from Merck (Darmstadt, Germany). Water (18.2 MΩ·cm, TOC < 5 ppb) was deionized and ultrapurified in a Milli-Q Plus by Merck Millipore (Bedford, MA, USA).

### 3.2. The HPLC-MS Apparatus, Conditions, and Software

Measurements were carried out using the UltiMate 3000 RS liquid chromatography system (Dionex, Sunnyvale, CA, USA) coupled with a MicrOTOF-Q II mass spectrometer with a time-of-flight mass analyzer (Bruker, Bremen, Germany) equipped with an autosampler and a thermostated Hypersil Gold Phenyl column (50 mm × 2.1 mm I.D., particles 1.9 µm). The mobile phase consisted of 0.1% formic acid in ultrapure water (A) and acetonitrile (B). The following gradient program (B) at a flow rate of 0.3 mL/min was used—0 min 15%; 4 min 40%; 7 min 40%; 10 min 70%, 12.5 min 15%; 17 min 15%—at a temperature of 35 °C, and the injection volume was 5 µL.

The chromatographic system was coupled with a mass spectrometer equipped with a positive ion electrospray ionization source (ESI) and MicroTOF-Q II, including a quadrupole analyzer combined with a subsequent collision cell and a time-of-flight analyzer (Bruker, Bremen, Germany). Identification of particular substances was performed based on the intensity of the received signals in the range covering the [M + H]^+^ values of target analytes (approximately 237–337 *m*/*z*) and their retention times. The parameters chosen as optimal for the MS analysis were capillary voltage: 4.5 [kV], nebulizer pressure: 2.5 bar, dry gas flow: 5.5 [L/min], and dry gas temperature: 200 °C.

Furthermore, each measurement sequence was performed by a TOF detector calibration using 10 mM sodium formate clusters in isopropanol and a water mixture (1:1, *v*/*v*) according to the Bruker procedure. The calibration solution was supplied to the MS device with the use of a syringe pump, which enabled the performing of independent chromatographic analyses.

Data were acquired and processed using software such as Chromeleon 6.8 (Dionex, Sunnyvale, CA, USA), HyStar 3.2, microTOFcontrol, and Compass DataAnalysis 4.0 (Bruker, Daltonics, Bremen, Germany), respectively. Compass IsotopePattern software version 4.1 (Bruker, Daltonics, Bremen, Germany) was also used to calculate the masses of [M + H]^+^ ions of all analytes, to obtain chromatograms of extracted ions.

### 3.3. DI-SPME Procedure

**Figure 4:**
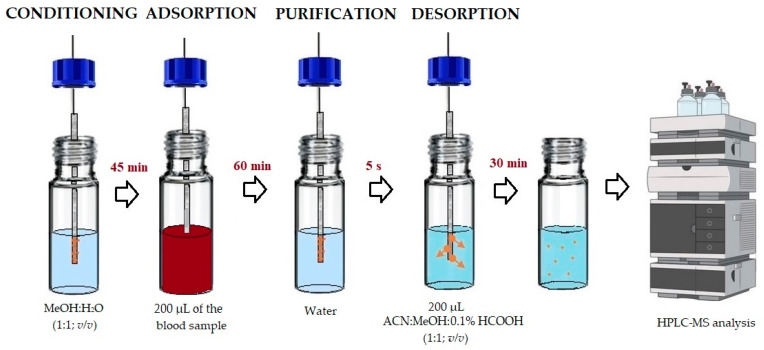
Scheme of the applied DI-SPME procedure.

The SPME procedure used in this work contains several steps which are presented in Figure 4 and the description below.

#### 3.3.1. Conditioning

Before placing the fibers in the tested solution, in the first step, they were conditioned in 1.5 mL of methanol: water (1:1, *v*/*v*) solution with continuous shaking at 2200 rpm for 45 min. The fibers prepared in this way were ready to adsorb the analytes.

#### 3.3.2. Adsorption

Because SPME is an equilibrium technique and analyte sorption is strictly monitored depending on the establishment of the interfacial equilibrium, it is necessary to determine the optimal equilibrium time between the number of analytes bound to the fiber and its concentration in the solution. Referring to the procedure of Majda et al. [12], the achievement of the equilibrium between phases of all tested analytes should take place after 60 min of adsorption at 25 °C with stirring at 2200 rpm, and these conditions were applied. The use of inserts allowed the sample volume to be minimized, so the adsorption process was performed with the use of only 200 μL of the blood sample. The inserts with the sample were placed in 1.5 mL HPLC vials.

#### 3.3.3. Purification

Taking into account the complexity of the biological sample and the content of high-molecular-weight components, the extraction process may be complicated. The mentioned macromolecules may cause, for example, co-extraction and formation of difficult-to-remove clots. To address this problem, we developed the two-step post-adsorption purification. Due to the lack of clots during the analysis performed, wiping the fibers with a dust-free tissue was skipped. Fibers were only washed in ultrapure water for 5 s with vortex agitation (5000 rpm).

#### 3.3.4. Desorption

According to the adopted procedure, solvent desorption in acetonitrile: methanol:0.1% formic acid solution (2:2:1, *v*/*v*/*v*), solution was used and it was carried out for 30 min at 25 °C with a stirring at 2200 rpm. Because of the use of inserts, the amount of desorption solution was minimized, and, thus, the evaporation time of the sample was shortened. After the desorption process was completed, the contents of the inserts were evaporated using a vacuum evaporator at 45 °C. After evaporation, 50 μL of 0.1% HCOOH as a mobile phase was added to the vials. The contents of the vials were then vortexed at a speed of 2500 rpm. The samples prepared in this way were ready for further LC-MS analysis.

#### 3.3.5. Fiber Cleaning

To enable the reuse of SPME fibers, after the analysis, residues of adsorbed analytes and other contaminants must be removed. For purification, the fibers were placed in 1.5 mL of the solution of methanol: water: isopropanol (2:2:1, *v*/*v*/*v*) stirred for 1 h at 2200 rpm, and, then, the fibers were left in this solution for 24 h.

### 3.4. Validation Procedure

To validate the newly developed SPME/LC-MS method, we followed the guidelines provided by the European Medicines Agency to ensure reliability [18]. The validation process involved determining a number of key parameters, including linearity, the limit of detection (LOD), the lower limit of quantification (LLOQ), intraday and interday precision, carryover, and dilution, as well as selectivity. Validation was performed only for drugs most commonly used in pharmacotherapy—AMI, CIT, FLU, PAR, SER, TRA, DUL, MIR, VEN, LAM, QUE, and OLA.

To calibrate the method, an interpolative internal standard approach was used. To generate calibration curves, a linear regression of ratios between the analytes peak area and the corresponding internal standard peak area (IA/IIS) was performed. The regression was carried out in a concentration range of 25, 50, 100, 150, 200, 250, and 300 ng/mL. Deuterated compounds that were used are shown in Table 1. The concentration of each IS (added to the samples before the extraction process) was constant and equal to 150 ng/mL. It was assumed that the coefficient of determination (R^2^) was not less than 0.99.

The limit of detection (LOD) is the smallest amount or lowest concentration of a substance that a given analytical method can detect with a certain probability. The value of LOD is closely related to the signal-to-noise ratio (S/N), which was assumed to be three (S/N = 3) in the calculations for the concentration of the analytes. The lower limit of quantification (LLOQ) is the minimum concentration of the analytes in a sample that can be quantified accurately and precisely. To ensure reliable quantification, the signal of the LLOQ sample should be at least five times greater than the signal of a blank sample [18].

In this research, precision was expressed as the coefficient of variation (CV). Precision intraday was calculated based on the results obtained for three identical samples prepared during one day under the same conditions by one analyst. Precision interday was calculated based on the results obtained on three consecutive days for the three samples tested during the day. Each CV parameter was calculated for concentrations of 50, 150, and 250 ng/mL according to the below Equation (1):

(1) $$CV=\frac{s}{x_{am}}\cdot 100\%$$

where s—standard deviation, and x_am_—arithmetic mean. According to the validation protocol [18], CV should not exceed 15%, but, at the lowest concentration level, it should be less than 20%.

Carryover and dilution were examined by analyzing blank blood samples before and after the analysis of blood standard samples at a concentration of 300 ng/mL for all analytes. In the case of dilution, the samples were diluted two and four times in the final stage of the sample preparation (by adding 100 μL or 200 μL of the 100-times-diluted mobile phase).

The analysis of blood from different individuals can be affected by the matrix effect (ME), which is caused by various factors such as different blood groups or hematocrit. To calculate ME for each matrix lot, the peak area in the presence of the matrix was divided by the peak area in the absence of the matrix. This was carried out by analyzing a blank matrix spiked after extraction with analytes and a pure solution of the analytes. The IS-normalized ME should also be calculated by dividing the ME of the analytes by the ME of the IS. The CV results of ME should not be greater than 15% [18].

When developing a new method, it may also be necessary to investigate the extent of any interference caused by metabolites of the drugs or interference from possible co-administered medications [18]. Due to this, the research was checked through an analysis of a mixture of drugs most often used in the pharmacology of mood disorders, plus ketamine (KET) and flunitrazepam (FLUN) included in “date-rape drugs”. Each substance was introduced into the human blood sample at a concentration of 100 ng/mL and analyzed using the developed methodology DI-SPME/LC-MS.

### 3.5. Sample Collection

Human blood (drug-free) was provided by a local blood bank (Cracow, Poland). Blood samples from patients (38 probes) who were taking analyzed drugs were provided by the Department of Adult Psychiatry at the Jagiellonian University Medical College (by Bioethical Commission Approval no. 1072.6120.302.2018). Each patient agreed to participate in the study and completed a questionnaire in which they voluntarily provided information. According to questionnaires, the average age of women patients in the research group was 48.9 years, while the average age of men was 50.7. As many as 32 of 40 researched patients suffer from bipolar disorder, while 8 patients were diagnosed with depression. SER, DUL, VEN, and TRA are the most popular active substances in the medications taken in the researched group.

In Supplementary Materials, Table S1 provides a summary of the data, including the types of drugs, doses, and frequency of use for individual patient samples. All samples were stored at −20 °C.

## 4. Conclusions

Based on the experiment results, it can be confidently concluded that the DI-SPME/LC-MS methodology developed is capable of extracting and identifying twelve different drugs from multiple groups of compounds. As such, it can be used as an effective preliminary screening method. The methodology also boasts satisfactory parameters of a calibration curve, limit of detection, limit of quantification, selectivity, and variation coefficients for each drug.

Additionally, the DI-SPME/LC-MS methodology has numerous advantages. The usage of DI-SPME extraction has led to a decrease in the consumption of toxic solvents and the amount of sample tested while maintaining the high sensitivity of the determinations. Therefore, the DI-SPME method presents itself as an interesting alternative to other extraction techniques, such as microwave extraction or solid-phase extraction, which are commonly used to isolate analytes from biological samples with a complex matrix.

Due to its simplicity and short analysis time, this technique has great potential in monitoring the concentration of drugs, especially antidepressants, over time. The results obtained from analyzing real patient samples confirm the potential of the developed method and suggest that it can be used in routine clinical trials. This technique can also be used to verify whether the patient is following the pediatrician’s recommendations and using the prescribed pharmacotherapy.

In conclusion, after conducting extensive research, it can be confidently stated that the method we have developed is a groundbreaking approach that combines DI-SPME (direct immersion–solid-phase microextraction) with liquid chromatography. This approach was used for the very first time in a study of a specific group of drugs that are commonly used in the pharmacotherapy of affective disorders. This pioneering technique has proven to be highly effective and is likely to pave the way for further research in this field.

## Figures and Tables

**Table 1:** A list of all analytes determined in the tests along with the assigned internal standards and the monitored range of ion masses and obtained retention time. Abbreviations used: amitriptyline (AMI), aripiprazole (ARI), carbamazepine (CBZ), citalopram (CIT), clomipramine (CLOM), desipramine (DEZ), flunitrazepam (FLUN), fluoxetine (FLU), fluvoxamine (FLUV), imipramine (IMI), ketamine (KET), paroxetine (PAR), sertraline (SER), trazodone (TRA), duloxetine (DUL), mirtazapine (MIR), nortriptyline (NORT), venlafaxine (VEN), lamotrigine (LAM), quetiapine (QUE), olanzapine (OLA), amitrip-tyline_d3 (AMI_d3), fluoxetine_d6 (FLU_d6), paroxetine_d6 (PAR_d6), venlafaxine_d6 (VEN_d6), and carbamazepine_d10 (CBZ_d10).

Drug Abbreviation	[M + H]^+^	t_R_ [min] in Spiked Blood	Deuterated Analogue	[M + H]^+^	t_R_ [min] in Spiked Blood
Tricyclic antidepressants					
AMI	278.1903 ± 0.01	6.44 ± 0.02	AMI-d_3_	281.2092 ± 0.01	6.26 ± 0.01
NORT	264.1747 ± 0.01	6.29 ± 0.02	AMI-d3	281.2092 ± 0.01	6.26 ± 0.01
IMI	281.2012 ± 0.01	6.26 ± 0.01	VEN-d6	284.2491 ± 0.01	3.83 ± 0.03
DEZ	267.1856 ± 0.01	6.10 ± 0.02	AMI-d3	281.2092 ± 0.01	6.26 ± 0.01
CLOM	315.1622 ± 0.01	7.10 ± 0.03	FLUX-d6	316.1789 ± 0.01	6.65 ± 0.02
Selective serotonin reuptake inhibitors					
CIT	325.1722 ± 0.01	5.68 ± 0.02	PAR-d_6_	336.1877 ± 0.01	6.16 ± 0.02
FLU	310.1413 ± 0.01	6.67 ± 0.02	FLU-d_6_	316.1789 ± 0.01	6.65 ± 0.02
FLUV	319.1621 ± 0.01	6.04 ± 0.02	FLU-d_6_	316.1789 ± 0.01	6.65 ± 0.02
PAR	330.1494 ± 0.01	6.18 ± 0.03	PAR-d_6_	336.1877 ± 0.01	6.15 ± 0.02
SER	306.0807 ± 0.01	6.93 ± 0.02	FLU-d_6_	316.1789 ± 0.01	6.64 ± 0.02
Serotonin and norepinephrine reuptake inhibitors					
DUL	298.1256 ± 0.01	6.38 ± 0.02	VEN-d_6_	284.2491 ± 0.01	3.81 ± 0.03
MIR	266.1667 ± 0.01	2.03 ± 0.02	AMI-d_3_	281.2092 ± 0.01	6.26 ± 0.01
VEN	278.2110 ± 0.01	3.83 ± 0.03	VEN-d_6_	284.2491 ± 0.01	3.80 ± 0.03
TRA	372.1605 ± 0.01	4.91 ± 0.03	PAR-d_6_	336.1877 ± 0.01	6.15 ± 0.02
Anticonvulsants and atypical neuroleptics					
ARI	448.1548 ± 0.01	6.78 ± 0.02	PAR-d_6_	336.1877 ± 0.01	6.16 ± 0.02
CBZ	237.1021 ± 0.01	5.40 ± 0.02	CBZ-d_10_	247.1650 ± 0.01	5.34 ± 0.03
LAM	256.0165 ± 0.01	1.83 ± 0.02	CBZ-d_10_	247.1650 ± 0.01	5.34 ± 0.03
QUET	384.1733 ± 0.01	4.91 ± 0.03	PAR-d_6_	336.1877 ± 0.01	6.16 ± 0.02
OLA	313.1475 ± 0.01	0.87 ± 0.02	FLU-d_6_	316.1789 ± 0.01	6.65 ± 0.02
“Date rape drugs”					
FLUN	314.0935 ± 0.05	6.63 ± 0.02	FLU-d_6_	316.1789 ± 0.01	6.65 ± 0.02
KET	238.0993 ± 0.05	1.62 ± 0.03	CBZ-d_10_	247.1650 ± 0.01	5.34 ± 0.03

**Table 2:** Summary of the validation parameters for DI-SPME/LC-MS method in the context of determining drugs used in the pharmacotherapy of mood disorders.

Parameters	AMI	CIT	FLU	PAR	SER	TRA	DUL	MIR	VEN	LAM	QUE	OLA
Linearity [ng/mL]	LLOQ—300											
R^2^	0.9907	0.9966	0.9922	0.9979	0.9940	0.9937	0.9975	0.9904	0.9978	0.9950	0.9960	0.9985
LLOQ [ng/mL]	0.92	4.87	2.32	2.60	8.43	2.69	21.47	0.70	2.91	5.73	4.58	7.62
LOD [ng/mL]	0.18	0.97	0.46	0.52	1.69	0.54	4.29	0.14	0.58	0.37	0.92	1.52
Precision [%] Intraday *												
50 [ng/mL]	12.55	10.23	8.44	16.61	5.80	9.20	6.04	19.60	3.47	7.54	16.24	10.94
150 [ng/mL]	6.80	3.68	5.97	7.84	7.35	10.59	8.51	6.07	3.13	9.39	8.01	5.36
250 [ng/mL]	5.27	6.14	12.38	13.85	11.08	13.38	11.04	8.31	9.53	1.85	12.62	13.41
Precision [%] Interday **												
50 [ng/mL]	19.05	19.05	3.34	17.85	13.57	11.74	10.03	16.94	8.84	16.85	18.89	16.47
150 [ng/mL]	10.88	10.88	1.57	11.69	12.11	7.63	3.67	12.58	5.69	14.30	9.16	7.61
250 [ng/mL]	12.48	12.48	7.34	12.46	7.85	7.48	11.59	10.17	5.05	11.70	5.60	12.84
Matrix effect [%]	7.54	4.58	6.02	8.15	9.02	8.51	6.41	4.99	2.46	7.54	7.32	3.68

* intraday precision obtained for 3 identical samples prepared during 1 day under the same conditions by 1 analyst; *n* = 9. ** interday precision obtained on 3 consecutive days for the 3 samples tested during the day; *n* = 27.

## Data Availability

Data are contained within the article and Supplementary Materials.

## Supplementary Materials

The following supporting information can be downloaded at: https://www.mdpi.com/article/10.3390/molecules29030676/s1, Table S1: Summary of information used drugs, the dose, and dosage in the patient’s group; Figure S1: The structure of the analytes.
