# Supplementary material for: Direct Immersion–Solid Phase Microextraction for Therapeutic Drug Monitoring of Patients with Mood Disorders
Source: Molecules. 2024 Jan 31;29(3):676. doi: 10.3390/molecules29030676 (PMC10856736; doi:10.3390/molecules29030676)
Supplement: Supplementary file 1 [file molecules-29-00676-s001.zip › Table S1. Summary of information used drugs, the dose and dosage in the patients group.pdf]

Table S1. Summary of information used drugs, the dose and dosage in the patients group.

ID	Drug	Dose [mg]	Dosage	ID	Drug	Dose [mg]	Dosage
**P_1**	TRA	150	0-0-1	**P_20**	CIT	10	1-0-0
	VEN	75	1-0-0		QUE	200	0-0-1
**P_2**	SER	50	0-0-1	**P_21**	DUL	30	1-0-1
	TRA	150	2-0-0		FLU	10	1-0-1
**P_3**	VEN	75	1-0-0		TRA	150	0-0-1
**P_4**	SER	50	0-0-1	**P_22**	MIR	30	1-0-1
**P_5**	VEN	150	1-0-0	**P_23**	DUL	60	1-0-0
**P_6**	DUL	60	1-0-0	**P_24**	SER	50	1-0-0
	TRA	150	0-0-1		TRA	50	1-0-0
**P_7**	MIR	15	1-0-0	**P_25**	SER	50	1-0-0
	VEN	75	1-0-0		QUE	25	1-0-1
**P_8**	SER	100	1-0-0	**P_26**	OLA	5	1-0-2
	TRA	150	0-0-1		SER	50	1-0.5-0
**P_9**	DUL	30	1-0-1		TRA	300	0-0-0.5
**P_10**	SER	50	1-1-0	**P_27**	SER	50	2-0-0
	OLA	5	0-0-1	**P_28**	QUE	400	0-0-1
**P_11**	SER	50	1-1-0	**P_29**	VEN	150	1-0-0
**P_12**	DUL	60	1-0-0	**P_30**	OLA	5	0-0-1
	QUE	25	1-0-1		SER	100	1-0-0
**P_13**	DUL	30	1-0-1	**P_31**	FLU	10	2-0-0
	LAM	50	1-0-1		TRA	150	0-0-1
	TRA	300	0-0-0.5	**P_32**	SER	100	1-0-1
**P_14**	SER	50	1-0-0	**P_33**	SER	100	1-0-1
**P_15**	TRA	75	0-0-1.33	**P_34**	DUL	60	1-0-0
	WEN	75	2-0-0		OLA	5	1-0-1
**P_16**	MIR	30	0-0-1	**P_35**	DUL	30	1-0-1
	QUE	75	1-0-0	**P_36**	AMI	10	1-0-1
	VEN	150	1-0-0		LAM	100	1-0-2
**P_17**	SER	50	2-1-0	**P_37**	TRA	150	1-0-1
**P_18**	VEN	75	1-0-0		DUL	60	1-0-0
**P_19**	PAR	20	1-0-0	**P_38**	TRA	150	1-0-1
