# Supplementary figures and images for: Direct Immersion–Solid Phase Microextraction for Therapeutic Drug Monitoring of Patients with Mood Disorders
Source: Molecules. 2024 Jan 31;29(3):676. doi: 10.3390/molecules29030676 (PMC10856736; doi:10.3390/molecules29030676)

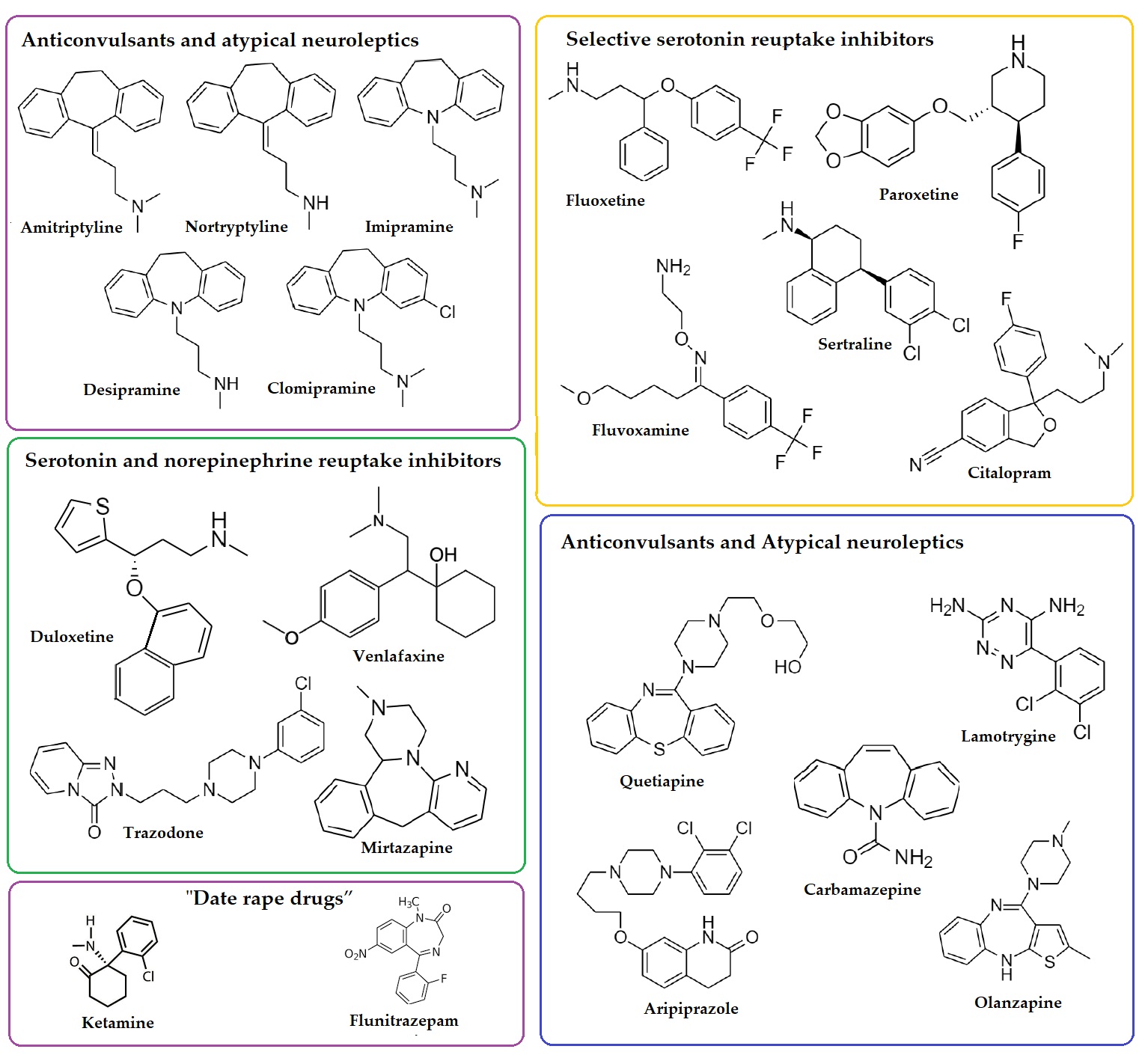

Supplement: Supplementary file 1 [file molecules-29-00676-s001.zip › Figure S1. The structure of the analytes.jpg]
